# The Potential of Microorganisms for the Control of Grape Downy Mildew—A Review

**DOI:** 10.3390/jof10100702

**Published:** 2024-10-08

**Authors:** Zhan-Bin Sun, Han-Jian Song, Yong-Qiang Liu, Qing Ren, Qi-Yu Wang, Xiao-Feng Li, Han-Xu Pan, Xiao-Qing Huang

**Affiliations:** 1 School of Light Industry Science and Engineering, Beijing Technology and Business University, Beijing 100048, China; twins5616@126.com (Z.-B.S.);; 2 State Key Laboratory for Biology of Plant Diseases and Insect Pests, Institute of Plant Protection, Chinese Academy of Agricultural Sciences, Beijing 100193, China

**Keywords:** *Plasmopara viticola*, grape Downy Mildew, biocontrol fungi, biocontrol bacteria

## Abstract

*Plasmopara viticola* (Berk.et Curtis) Berl. Et de Toni is the pathogen that causes grape downy mildew, which is an airborne disease that severely affects grape yield and causes huge economic losses. The usage of effective control methods can reduce the damage to plants induced by grape downy mildew. Biocontrol has been widely used to control plant diseases due to its advantages of environmental friendliness and sustainability. However, to date, only a few comprehensive reviews on the biocontrol of grape downy mildew have been reported. In this review, we summarize the biological characteristics of *P. viticola* and its infection cycle, followed by a detailed overview of current biocontrol agents, including bacteria and fungi that could be used to control grape downy mildew, and their control effects. Furthermore, potential control mechanisms of biocontrol agents against grape downy mildew are discussed. Lastly, suggestions for future research on the biocontrol of grape downy mildew are provided. This review provides the basis for the application of grape downy mildew biocontrol.

## 1. Introduction

As an important economic fruit, grapes possess significant nutritional value and are rich in minerals, vitamins, and amino acids. Grapes also hold crucial economic value and serve as primary sources in many local agricultural economies. However, grape cultivation is often influenced by a number of different diseases, including grape downy mildew, grape anthracnose, grape gray mold, grape white rot, and other diseases [1,2,3,4]. Among these grape diseases, grape downy mildew is a significant threat. Grape downy mildew is caused by the obligate parasitic oomycetes *Plasmopara viticola* (Berk.et Curtis) Berl. Et de Toni [5]. Infection with grape downy mildew can lead to reductions in grape yield, with severe cases resulting in 70–80% yield losses, or in some cases, no harvest produced [6]. In addition to impacting grape yields, grape downy mildew can also cause serious economic losses [7].

At present, methods for controlling grape downy mildew include chemical control, the development of resistant varieties, and biological control. Chemical control is a commonly used control method with the advantage of quickly and efficiently preventing the occurrence and development of grape downy mildew. Several pesticides, including azoxystrobin, dimethomorph, and picoxystrobin, have been reported to be involved in controlling grape downy mildew [8,9,10]. However, the use of chemical pesticides can cause severe environmental pollution. Moreover, the long-term use of pesticides can also enhance pathogens’ resistance to pesticides, which limits the application of chemical control agents. The development of disease-resistant grape varieties can improve the resistance of plants to grape downy mildew. A number of grape downy mildew-resistant varieties have been developed [11,12]. However, due to geographical differences in the cultivation of disease-resistant grape varieties, the development of disease-resistant varieties that are suitable for different geographical environments is limited. As a green and sustainable control method, biological control (biocontrol) has attracted considerable attention.

Biocontrol commonly refers to the control of plant diseases by using beneficial biological agents. Compared with chemical control, the advantage of biocontrol is that it does not pollute the environment, in addition to its sustainable usage. With regard to the biocontrol of plant diseases, several microorganisms, including bacteria and fungi, have exhibited excellent plant disease-control capabilities. Once biocontrol microorganisms are inoculated into plants or the surrounding soil, they can continue to prevent disease through their own growth and passage. *Bacillus* is the most widely reported bacterial agent used for biocontrol. Several *Bacillus* species—*B. subtilis*, *B. thuringiensis*, *B. velezensis*, *B. amyloliquefaciens*, and *B. aryabhattai*—exhibit effective control capabilities against different types of plant diseases [13,14,15,16,17]. Other bacterial species, such as *Pseudomonas*, *Streptomyces*, and Paenibacillus, are also reported as biocontrol agents against plant diseases [18,19,20]. *Trichoderma* is the most commonly reported species of biocontrol fungus. Species such as *T. harzianum*, *T.virens*, *T. atroviride*, *T. hamatum,* and *T. longibrachiatum* are reported to be involved in plant disease control [21,22,23,24,25]. In addition to *Trichoderma*, other fungal species such as *Clonostachys rosea*, *Coniothyrium minitans*, *Beauveria bassiana*, and *Metarhizium anisopliae* also act as biocontrol agents and play important roles in the control of plant diseases [26,27,28,29]. In addition to the biocontrol effect, many biocontrol microorganisms can also promote plant growth, thereby playing a dual effect in disease prevention and growth promotion [30,31].

Biocontrol microbial agents mainly exhibit their plant disease-control capabilities through the following mechanisms: competition for space and nutrients with pathogens, the secretion of enzymes such as cell wall-degrading enzymes, the production of metabolites such as toxins or antibiotics, inducing plant systemic resistance against plant diseases, and directly parasitizing the host pathogen in some special biocontrol agents. To date, a number of biocontrol microorganisms, such as *Bacillus* and *Trichoderma*, have been reported to be involved in controlling grape downy mildew, in addition to reports on their control mechanisms [32,33]. However, only a few systematic reviews on the biological control of grape downy mildew have been reported.

Therefore, in this review, we mainly focus on advances in the biocontrol of grape downy mildew. The biological characteristics and infection cycle of *P. viticola* are introduced first, in addition to the symptoms of grape downy mildew. Secondly, biocontrol agents currently employed against grape downy mildew are discussed. Thirdly, and most importantly, we provide a detailed introduction to the biocontrol agents, including bacteria and fungi, used for controlling grape downy mildew, and their control efficacy. Fourthly, the potential mechanisms of biocontrol agents in controlling grape downy mildew are discussed. Lastly, an analysis of the problems present in the biocontrol of grape downy mildew, along with corresponding countermeasures and potential further directions for grape downy mildew biocontrol, are discussed. This review provides the basis for the application of grape downy mildew biocontrol.

## 2. Biological Characteristics of *Plasmopara viticola*

In the asexual stage, *P. viticola* produces sporangiophores. The sporangium is generated on the top of the sporangiophore. The sporangium germinates to produce zoospores. At the end of the growing season of grape plants, *P. viticola* produces oospores through sexual reproduction. Oospores germinate and a germ tube is formed, from which the sporangium is generated at its top and zoospores are produced through germination [34,35].

**Figure 1:**
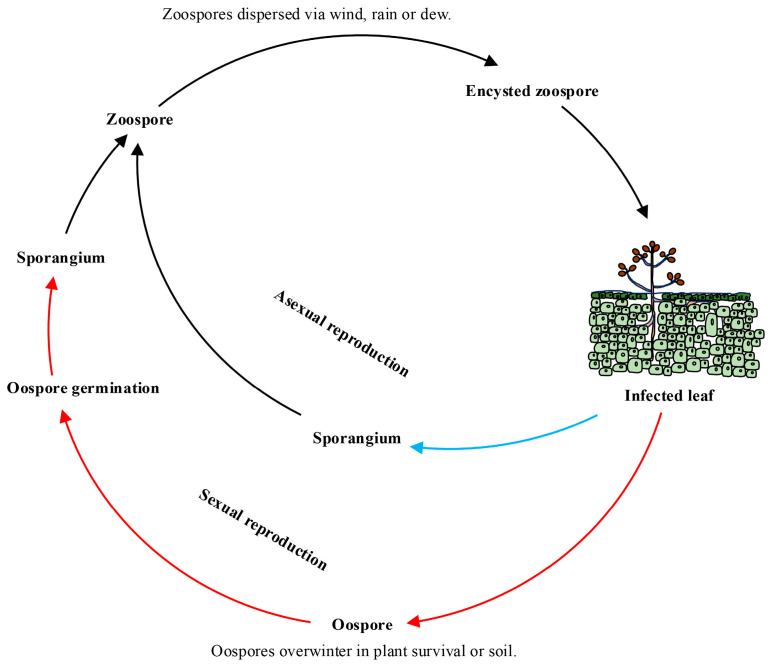
Infection cycle of *Plasmopara viticola*. The red arrow represents the direction and starting. point of the primary infection; the blue arrow indicates the direction and starting point of the secondary infection.

**Figure 2:**
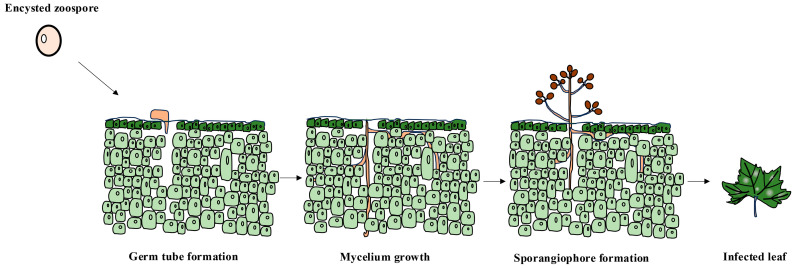
The biological process of *Plasmopara viticola* infecting grape plants.

The occurrence of grape downy mildew is affected by temperature and humidity [36]. Oospores of *P. viticola* exhibit high stress resistance and can overwinter in plant survival or soil. They can germinate in water or moist soil to form sporangia, releasing zoospores under suitable conditions. Zoospores can disperse to grape leaves via wind, rain, or dew and then invade through stomata, resulting in primary infection [37]. After infecting grape leaves, *P. viticola* forms mycelia, which can propagate and spread in the intercellular spaces of grape tissue cells and enter grape leaf cells to absorb nutrients through the haustorium. The sporangiophore and sporangium appear at grape leaf disease spots. Sporangium can germinate and release zoospores, which are spread by wind and rain to cause the secondary infection of grape downy mildew. The infection process of *P. viticola* in grapes mainly involves three stages: sporangium germination, zoospore release, and invasion into grape tissue cells. During the entire growing season of grapes, if environmental conditions are favorable, *P. viticola* can carry out multiple repeated infections of grape leaves, resulting in the severe epidemic of grape downy mildew. At the end of the grape growth stage, *P. viticola* forms oospores during the sexual reproduction stage, which are the primary infection source in the following year [38,39,40] (Figure 1 and Figure 2).

*P. viticola* can infect any green young tissue of grapes, among which young leaves are most susceptible to infection [41]. During the early infection stage, young, infected leaves exhibit light-yellow and watery lesions with unclear edges. Thereafter, the lesions expand rapidly and gradually develop into brown, irregular or polygonal spots.

In severe cases, several spots merge to form large, irregular spots. Under humid weather conditions, the sporangiophore and sporangium of the *P. viticola* are formed on the back surface of the lesions and appear as a white, frosty mildew layer, which is the most obvious feature of grape downy mildew. Apart from young leaves, other tissues such as the young tips, inflorescence, stems, or fruits of grapes can also easily be infected by *P. viticola* under high-humidity conditions, resulting in a white mildew layer in the lesions [42,43,44,45]. Because *P. viticola* is capable of infecting different types of grape tissues, causing huge damage to the production of grapes, understanding the molecular mechanisms of *P. viticola* infection in grape plants is therefore of great significance for exploring potential targets for its control and further improving the control efficacy of grape downy mildew.

Effector-encoding genes have been widely studied in *P. viticola* infection. In a study, the results of a transcriptomic analysis revealed that multiple effector-encoding genes were highly differentially expressed in *P. viticola* following inoculation on a grapevine cultivar [46]. PvRxLR18 and PvRxLR28 effector genes were strongly upregulated in a high-virulence *P. viticola* strain compared to a low-virulence strain [47]. The RxLR effector-encoding gene PvAvh77 was strongly upregulated during the initial stage of *P. viticola* infection in grape plants [48]. In addition to RXLR effector-encoding genes, carbohydrate-active enzymes and pathogenicity genes have also been found to be significantly upregulated during *P. viticola* infection [49].

## 3. Biocontrol Agents

### 3.1. Screening Biocontrol Agents

Because *P. viticola* is an obligate parasite, it is not possible to cultivate it on artificial media [49]. Therefore, the screening of biocontrol microorganisms for controlling grape downy mildew mainly relies on leaf disk, detached leaf, greenhouse or pot, and field assays. In particular, preliminary screening cannot be carried out on artificial media, which increases the overall workload. Therefore, in order to improve the efficiency of preliminary screening, fungi that are closely related to *P. viticola* were selected as target pathogens for preliminary screening, such as *Phytophthora* sp., which belongs to the same family (Peronosporaceae) as *P. viticola*. Guo et al. [50,51] isolated 153 bacteria and 87 fungi from grapevine leaves and used *Phytophthora capsici* as the target pathogen for preliminary screening. Lastly, five bacteria and four fungi with evidently antagonistic effects on *Phytophthora capsica* were screened, with their *P. viticola* control capabilities further evaluated using the leaf disk method.

Moreover, traditional screening methods for biocontrol agents involve initially isolating numerous strains from the soil, rhizosphere, or grape plants and then evaluating the potential biocontrol capabilities of the isolated strains. Although biocontrol agents capable of controlling grape downy mildew can eventually be obtained through this method, the workload is extremely high. A more efficient screening method is isolating potential biocontrol agents from healthy grapevine tissues from grape plants that are severely diseased by grape downy mildew. This effect is mainly due to grape downy mildew being an airborne disease and the incidence among the same grape variety being similar. Therefore, microorganisms with potential control capabilities against grape downy mildew might exist in healthy grape tissues surrounded by severely infected plants. The use of this method can improve the efficiency of screening biocontrol agents against grape downy mildew. Liu et al. [52] isolated 56 endophytic bacteria from healthy grapevine leaves in a grape downy mildew-infected vineyard and found nine bacteria with biocontrol capabilities against grape downy mildew using the detached leaf method.

There are a number of types of biocontrol microorganisms; however, only a few biocontrol agents, including bacteria (e.g., *Bacillus* sp., *Streptomyces* sp., *Pseudomonas* sp., *Paenibacillus*, and *Lysobacter* sp.) and fungi (e.g., *Trichoderma* sp., *Beauveria* sp., *Fusarium* sp., *Penicillium* sp., and *Saccharomyces* sp.), exhibit strong control capabilities against grape downy mildew.

### 3.2. Bacteria as Control Agents for Grape Downy Mildew

*Bacillus* and *Streptomyces* are the main biocontrol bacteria exhibiting a strong ability to control grape downy mildew. Multiple *Bacillus* species, including *B. subtilis*, *B. pumilus*, *B. megaterium*, *B. velezensis*, *B. amyloliquefaciens*, and *B. methylotrophicus*, have been reported to be involved in the control of grape downy mildew (Table 1).

*B. subtilis* are the most widely reported *Bacillus* species in controlling grape downy mildew. In a study, *B. subtilis* GLB191 was isolated from grapevine leaves and exhibited a dramatic reduction in the number of infection sites in *P. viticola* zoospores through the use of its supernatant. Field assay results demonstrated that the spraying of GLB191 could significantly decrease grapevine disease severity caused by *P. viticola* [53]. The results of in-depth studies showed that the biocontrol ability of GLB191 is mainly attributed to inducing the plant defense response and the production of metabolites. The application of the supernatant of GLB191 improves the expression level of plant defense response-related genes in grape plants, including genes encoding stilbene synthase, PR protein 3 chitinase 4c, and PR protein 2 β-1,3-glucanase. Moreover, GLB191 is able to produce the cyclic lipopeptides fengycin and surfactin. The disruption of fengycin and surfactin synthesis-encoding genes in GLB191 mutants results in a decrease in the amount of fengycin and surfactin in the supernatant. The sporulation of GLB191 mutants was significantly higher than in the wild strain [54]. Other *B. subtilis* strains, such as KS1 isolated from grape berry skins, B-FS01 isolated from oilseed rape stalk, and HMB-20428 isolated from grapevine leaves, have also exhibited excellent biocontrol efficiency against grape downy mildew under field conditions [55,56,57].

In addition to *B. subtilis*, other *Bacillus* species have shown biocontrol capabilities against grape downy mildew. *B. megaterium* BMJBN02 was isolated from leek farmland soil. Spraying plants with BMJBN02 can significantly decrease the incidence rate and disease index of grape downy mildew, with a similar efficiency to 0.1% nicotinyl morpholine. The results of in-depth studies showed that BMJBN02 could induce a defense response in grape plants, resulting in dramatically increased salicylic acid content, as well as higher expression of the plant defense response-related genes PR1, PR2, PR5, and PR10.2 in grape plants through the application of BMJBN02 [58]. Similarly, another *Bacillus* species, *B. velezensis* KOF112, isolated from a grapevine shoot xylem, is also able to induce a plant defense response. Genes encoding class IV chitinase and β-1,3-glucanase were significantly upregulated in grape leaves following the usage of KOF112. Furthermore, KOF112 was found to be able to reduce the disease severity of grape downy mildew through the leaf disk assay and inhibit the zoospore release of *P. viticola* [59].

*B. amyloliquefaciens* strains YTB1407 and CS5, isolated from American ginseng root and grapevine leaf, respectively, showed strong control capabilities against grape downy mildew [52,60]. Another *B. amyloliquefaciens* strain, N22, exhibited a control efficiency of 76.94% against grape downy mildew under field conditions [61]. Kang et al. isolated *B. methylotrophicus* T3 from soil and completely inhibited the incidence of grape downy mildew through the use of the detached leaf assay [62].

In addition to *Bacillus* strains, *Streptomyces* strains also play an important role in controlling grape downy mildew. In a study, *S. atratus* PY-1, isolated from soil, exhibited a strong ability to reduce disease severity caused by *P. viticola* in both the detached leaf (92.13%) and field assay (83%). The results of in-depth studies showed that the fermentation culture of PY-1 could damage the sporangia and sporangiophores of *P. viticola*. Moreover, 5-acetoxycycloheximide and cycloheximide were purified from PY-1, showing effective capabilities against *P. viticola* [63]. Khatmiamycin, a novel compound derived from *Streptomyces* sp. ANK313, has complete motility inhibitory and lytic effects (83 ± 7%) on zoospores of *P. viticola* [64]. *S. microflavus* QH94 and *S. lydicus* A02, isolated from soil, displayed control efficiencies of 89.8% and 95.7% against grape downy mildew, respectively [65,66]. In addition, the supernatant of *S. corchorusii* NF0919 effectively inhibited the spore germination of *P. viticola* [67]. The application of *S. viridosporus* HH1 and *S. violates* HH5 could significantly reduce the disease severity of grape downy mildew [68].

*Pseudomonas aeruginosa* HB135 and *Ps. azotoformans* L-B-4, isolated from leaves, showed 91.27% and 97.2% control efficiency, respectively, against grape downy mildew when the detached leaf assay was used [69]. Additionally, L-B-4 was found to inhibit the sporangium germination of *P. viticola* [70]. Lakkis et al. isolated *P. fluorescens* PTA-CT2 from a healthy, field-grown grapevine and found that PTA-CT2 could dramatically reduce the disease severity of grape downy mildew under greenhouse conditions. The results of in-depth studies showed that the main control mechanism of PTA-CT2 is the induction of plant systemic resistance [71].

*Paenibacillus polymyxa* PB-2, together with its sterile filtrate, was found to be able to strongly inhibit *P. viticola* [72]. Hao et al. reported that another *Paenibacillus* sp. strain, B2, could induce the expression of plant defense genes, such as PR-3 (proteinase inhibitor), CHI (chitinase), and LOX (lipoxygenase). An antagonistic peptide, paenimyxin was purified from B2 with the ability to inhibit zoospore motility and reduce the number of infection sites of *P. viticola* [73]. *Microbacterium testaceum* N6 and *Ochrobactrum* sp. SY286 were both isolated from grapevine leaves and exhibited strong control capabilities against grape downy mildew [50,74]. The fermentation fluid of SY286 caused distortions in the mycelium and crimpled or ruptured the sporangium of *P. viticola* [75]. *Lysobacter capsic* AZ78 was isolated from the tobacco rhizosphere and showed equal control efficiency to fungicides in controlling grape downy mildew under field conditions [76,77].

**Table 1:** Overview of biocontrol bacteria control grape downy mildew.

Biocontrol Microorganisms	Strain Name	Isolation Source	Application Scale	Application Manner	Application Types	Application Concentration	Control Efficiency
*Ochrobactrum* sp.	SY286 [74]	Grapevines leaves	Field	Spray	Live organisms	10^8^ cfu/mL	91.05%
*Paenibacillus polymyxa*	PB-2 [72]	-	Field	Spray	Live organisms	10^9^ cfu/g	46.91%
*Paenibacillus* sp.	B2 [73]	Sorghum mycorrhizosphere	Leaf discs	-	Metabolites	0, 5, 10, 50 μg/mL	-
*Pseudomonas aeruginosa*	HB135 [69]	Grapevines leaves	Detached leaf	-	Live organisms	10^7^ cfu/mL	91.27%
*Pseudomonas fluorescens*	PTA-CT2 [71]	Healthy filed-grown grape-vine	Greenhouse	Spray	Live organisms	10^7^ cfu/mL	-
*Pseudomonas azotoformans*	L-B-4 [70]	Grapevines leaves	Detached leaf	-	Live organisms	1 × 10^8^ cfu/mL	97.2%
*Microbacterium testaceum*	N6 [50]	Grapevines leaves	Leaf discs	-	Live organisms	1 × 10^7^ cfu/mL	74.2%
*Lysobacter capsici*	AZ78 [76]	Tobacco rhizosphere	Field	Spray	Live organisms	10^8^ cfu/mL	-
*Streptomyces atratus*	PY-1 [63]	Soil	Field	Spray	Fermentation solution	-	90.08%
*Streptomyces microflavus*	QH94 [65]	Soil	Field	Spray	Fermentation solution	-	82.53%
*Streptomyces lydicus*	A02 [52]	Soil	Field	Spray	Metabolites	-	95.7%
*Streptomyces viridosporus*	HH1 [68]	-	-	Spray	Live organisms	3 × 10^7^/mL	-
*Streptomyces violatus*	HH5 [68]	-	-	Spray	Live organisms	3 × 10^7^/mL	-
*Streptomyces corchorusii*	NF0919 [67]	-	-	Spray	Fermentation supernatant	-	71.55%
*Streptomyces* sp.	ANK313 [64]	Soil	-	-	Metabolites	-	-
*Bacillus subtilis*	KS1 [55]	Grape berry skins	Field	Spray	Live organisms	1 × 10^8^ cells/mL	-
*Bacillus subtilis*	GLB191 [53]	Grapevines leaves	Field	Spray	Live organisms	10^8^ cfu/mL	-
*Bacillus subtilis*	B-FS01 [56]	Oilseed rape stalk	Field	Spray	Live organisms	10^7^/mL	88.25%
*Bacillus subtilis*	HMB-20428 [57]	Grapevines leaves	Field	Spray	Live organisms	1 × 10^8^ cfu/mL	54.66%
*Bacillus subtilis*	JL4 [78]	Grapevines leaves	Greenhouse	Spray	Live organisms	8 × 10^8^ cfu/mL	88%
*Bacillus subtilis*	DJ-6 WP [67]	-	Field	Spray	Live organisms	1 × 10^11^ cfu/g	70.71%
*Bacillus pumilus*	GLB197 [53]	Grapevines leaves	Field	Spray	Live organisms	10^8^ cfu/mL	-
*Bacillus megaterium*	BMJBN02 [58]	Soil	Plot	Spray	Metabolites crude extract	-	-
*Bacillus velezensis*	KOF112 [59]	Grapevine shoot xylem	Leaf discs	-	Live organisms	1 × 10^8^ cfu/mL	100%
*Bacillus amyloliquefaciens*	YTB1407 [60]	American ginseng root	Leaf discs	-	Fermentation liquor	-	58.05%
*Bacillus amyloliquefaciens*	N22 [61]	-	Field	Spray	Live organisms	1 ×10^7^/mL	76.94%
*Bacillus amyloliquefaciens*	CS5 [53]	-	Detached leaf	-	Live organisms	10^8^ cfu/mL	96.23%
*Bacillus methylotrophicus*	T3 [62]	Soil	Field	Spray	Live organisms	2 × 10^8^ cfu/mL	55.4%
*Bacillus* sp.	BCJB01 [79]	-	Field	Spray	Live organisms	3 × 10^9^ cfu/mL	84.05%

Note: “-” represents not applicable.

### 3.3. Fungi as Control Agents for Grape Downy Mildew

*Trichoderma* and *Fusarium* are the main biocontrol fungi with the ability to control grape downy mildew. *T. harzianum* is the most widely reported *Trichoderma* species involved in grape downy mildew biocontrol. Banani et al. [80] found that the application of *T. harzianum* T39 significantly reduces the disease severity of grape downy mildew, in addition to improving the expression levels of defense-related genes, such as pathogenesis-related protein 2 (PR-2), pathogenesis-related protein 4 (PR-4), and osmotin 1 (OSM-1) in grape plants. Similar findings have been reported in *T. harzianum* TriH_JSB36, which elicited defense responses in grape plants and enhanced the activity of defense-related enzymes, including 1,3-glucanase, peroxidase, and phenylalanine ammonia-lyase [81]. In a study, the application of *T. harzianum* HL1 and HL14 led to a dramatic decrease in the disease severity of grape downy mildew [68,82]. Other *Trichoderma* species, such as *T. viride* HL5, have been found to be able to significantly increase the activity of oxidative enzymes and contain peroxidase and polyphenol oxidase [68]. The usage of *T. asperellum* ICC012 and *T. gamsii* ICC080 triggered the production of jasmonic acid, which is an important factor in inducing plant defense response [83].

*Fusarium proliferatum* strain G6 was isolated from atypical grape downy mildew lesions and significantly reduced the disease severity of grape downy mildew under field conditions. Through microscope observations, it was found that G6 could coil around and inside the sporangiophores of *P. viticola* [84]. Another *F. proliferatum* strain, F3, isolated from abnormal lesions of grape downy mildew, displayed strong control capabilities against grape downy mildew when the detached leaf assay was used. The conidial suspension and sterile fermentation fluid of F3 showed 86.8% and 83.1% inhibition rates for the sporangium germination of *P. viticola*. Microscope observation demonstrated that F3 could coil around or cover the sporangiophores of *P. viticola* [85]. Other *Fusarium* species, including *F. delphinoides* M1, *F. brachygibbosum* M2, *F. pseudonygamai* M10, *F. pseudonygamai* M12_1, and *Fusarium* sp. M12_2, isolated from the sporangiophore of *P. viticola*, could lyse and inhibit the production of sporangia in *P. viticola*. The metabolite fusaric acid, produced by these Fusarium species, could inhibit the growth of *P. viticola* [86,87].

Some fungal species exhibit their biocontrol capabilities through the production of metabolites. An aqueous extract from *Penicillium chrysogenum*, known as Pen, was found to significantly reduce the disease severity of grape downy mildew under field conditions [88]. *Phomopsis* sp. CAFT69 was isolated from the leaves and stem bark of *Endodesmia calophylloides*, producing excelsional, 9-hydroxyphomopsidin, and alternariol, which could inhibit the motility and lysis of *P. viticola* zoospores [89]. Musetti et al. [90,91] found that *Alternaria alternata* is able to produce three diketopiperazine metabolites—cyclo (L-leucine-trans-4-hydroxy-l-proline), cyclo (L-phenylalanine-trans-4-hydroxy-l-proline), and cyclo (L-alanine-trans-4-hydroxy-l-proline)—for the complete control of grape downy mildew.

Some fungal species can induce grape plant resistance to *P. viticola* infection. Four different sources of *Beauveria bassiana,* strains—ATP01, ATP05, EABb 04/01-Tip—and ATCC 74040 were found to be able to significantly reduce the severity of grape downy mildew. The results of in-depth studies showed that the application of strain ATCC 74040 could increase the expression level of the defense-related PR-1-like gene in grape plants [92,93]. Moreover, *Pythium oligandrum* Po37 isolated from grapevine roots could induce the expression of genes from the jasmonate and ethylene pathways when the grape plants were infected with *P. viticola* [94]. *Rhizophagus irregularis* AMF could alter the expression level of PvRxLR28, a pathogenicity effector of *P. viticola*, thereby reducing the ability of *P. viticola* to infect grape plants [95].

Various fungal species, such as *Acremonium byssoides* A21, isolated from grapevine leaves, were able to completely inhibit the sporangia of *P. viticola* when the culture filtrates and crude extract of A21 were used [96]. Other *Acremonium* species, such as *A. sclerotigenum* A59 and *A. persicinum* A3, exhibited high inhibition rates against the sporangia germination of *P. viticola* [97]. In a study involving *Epicoccum nigrum* isolated from the surface of *P. viticola*-infected leaves, it was observed that the mycelia were surrounded by sporangiophores of *P. viticala* [98]. *Leptosphaerulina australis* Y29, isolated from grapevine leaves, displayed a control efficiency of 72.9% against grape downy mildew when the leaf disk assay was used [51]. In addition, the usage of *Aureobasidium pullulans* could reduce the number of lesions on leaves caused by *P. viticala* compared with the control [99]. It has also been reported that the yeast strain *Saccharomyces cerevisiae* is able to significantly reduce the disease severity of grape downy mildew [100] (Table 2).

**Table 2:** Overview of biocontrol fungi control grape downy mildew.

Biocontrol Microorganisms	Strain Name	Isolation Source	Application Scale	Application Manner	Application Types	Application Concentration	Control Efficiency
*Acremonium byssoides*	A21 [96]	Grapevines leaves	-	-	Culture filtrates, crude extracts	-	-
*Acremonium sclerotigenum*	A59 [97]	Grapevines seed	-	-	Culture filtrates	-	-
*Acremonium persicinum*	A3 [97]	Grapevines leaves	-	-	Culture filtrates	-	-
*Penicillium chrysogenum*	- [88]	-	Field	Spray	Metabolites	45 g/L	90%
*Alternaria alternata*	- [90]	Grapevines leaves	Greenhouse	Spray	Metabolites	10^−3^, 10^−4^, 10^−5^, 10^−6^ M	100%
*Fusarium delphinoides*	M1 [86]	*P. viticola* sporangiophore	Leaf discs	-	Culture extract	10 μg/mL	-
*Fusarium brachygibbosum*	M2 [86]	*P. viticola* sporangiophore	Leaf discs	-	Culture extract	10 μg/mL	-
*Fusarium pseudonygamai*	M10 [86]	*P. viticola* sporangiophore	Leaf discs	-	Culture extract	10 μg/mL	-
*Fusarium pseudonygamai*	M12_1 [86]	*P. viticola* sporangiophore	Leaf discs	-	Culture extract	10 μg/mL	-
*Fusarium* sp.	M12_2 [86]	*P. viticola* sporangiophore	Leaf discs	-	Culture extract	10 μg/mL	-
*Fusarium proliferatum*	G6 [84]	Atypical grape downy mildew lesions	Field	Spray	Live organisms	1 × 10^6^ microconidia/mL	-
*Fusarium proliferatum*	F3 [85]	Abnormal lesions of grape downy mildew	Detached leaf	-	Live organisms	1.7 × 10^7^ spores/mL	88.9%
*Pythium oligandrum*	Po37 [94]	Grapevine roots	Foliar discs	-	Live organisms	2 × 10^4^ oospores/mL	-
*Beauveria bassiana*	ATP01 [94]	Maize stem borer	Greenhouse	Spray	Live organisms	1 × 10^8^ conidia/mL	-
*Beauveria bassiana*	ATP05 [92]	Sorghum chafer	Greenhouse	Spray	Live organisms	1 × 10^8^ conidia/mL	-
*Beauveria bassiana*	EABb04/01-Tip [92]	Dead *Timaspis papaveris* (Kieffer) larvae	Greenhouse	Spray	Live organisms	1 × 10^8^ conidia/mL	-
*Beauveria bassiana*	ATCC 74040 [92]	-	Greenhouse	Spray	Live organisms	1.4 × 10^7^ conidia/mL	89.03%
*Saccharomyces cerevisiae*	- [100]	-	-	Spray	Live organisms	1.5 L/ha	-
*Saccharomyces cerevisiae*	- [68]	-	-	Spray	Live organisms	1 × 10^9^ cell/mL	-
*Aureobasidium pullulans*	- [99]	-	Greenhouse	Spray	Live organisms	1.0%	-
*Leptosphaerulina australis*	Y29 [51]	Grapevines leaves	Leaf discs	-	Live organisms	1 × 10^7^ spores/mL	72.9%
*Epicoccum nigrum*	- [98]	*P. viticola* infected leaves	-	-	Live organisms	-	-
*Phomopsis* sp.	CAFT69 [89]	Leaves or stems bark of *Endodesmia calophylloides*	-	-	Metabolites	10, 30, 50 μg/mL	-
*Rhizophagus irregularis*	- [95]	-	-	-	Live organisms	-	-
*Trichoderma harzianum*	TriH_JSB36 [81]	Soil	Field	-	Live organisms	10^8^ spores/mL	82.9%
*Trichoderma harzianum*	HL1 [68]	Soil	-	Spray	Live organisms	3 × 10^7^/mL	-
*Trichoderma harzianum*	HL14 [82]	Bean rhizosphere	Field	Spray	Live organisms	10^8^ spores/mL	69.7%
*Trichoderma harzianum*	T39 [80]	-	Greenhouse	Spray	Live organisms	1 × 10^7^ conidia/mL	-
*Trichoderma viride*	HL5 [68]	Soil	-	Spray	Live organisms	3 × 10^7^/mL	-
*Trichoderma asperellum*	ICC012 [83]	-	Laboratory	Spray	Live organisms	2.5 kg/hl	-
*Trichoderma gamsii*	ICC080 [83]		Laboratory	Spray	Live organisms	2.5 kg/hl	-

Note: “-” represents not applicable.

## 4. Mechanisms of Action of Biocontrol Agents against *Plasmopara viticola*

**Figure 3:**
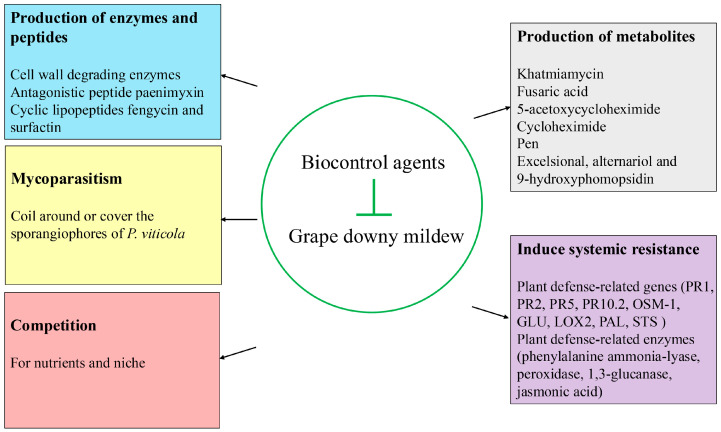
Mechanisms of biocontrol agents against grape downy mildew.

Several mechanisms are involved in biocontrol agents’ control of grape downy mildew, including the production of metabolites, the secretion of enzymes, inducing plant systemic resistance in plants, mycoparasitism, and competition (Figure 3, Table 3).

**Table 3:** Mechanisms of biocontrol microorganisms control grape downy mildew.

Biocontrol Microorganisms	Mechanisms	References
*Beauveria bassiana* ATCC74040	Competition; induce the expression of defense-related gene PR-1-like in grape plants	[92]
*Pythium oligandrum* Po37	Induce the expression of defenses genes (PR1, GLU, PR5, LOX2, PAL and STS)	[94]
*Fusarium proliferatum* F3	Mycoparasitism	[85]
*Fusarium proliferatum* G6	Mycoparasitism	[84]
*Fusarium delphinoides* M1	Mycoparasitism; production of metabolites, fusaric acid, etc.	[86,87]
*Fusarium brachygibbosum* M2	Mycoparasitism; production of metabolites, fusaric acid, etc.	[86,87]
*Fusarium pseudonygamai* M10	Mycoparasitism; production of metabolites, fusaric acid, etc.	[86,87]
*Fusarium pseudonygamai* M12_1	Mycoparasitism; production of metabolites, fusaric acid, etc.	[86,87]
*Fusarium* sp. M12_2	Mycoparasitism; production of metabolites, fusaric acid, etc.	[86,87]
*Alternaria alternata*	Production of three dipeptides, belonging to the family of diketopiperazines; competition	[90,91]
*Penicillium chrysogenum*	Production of Pen, an aqueous extract of the dry mycelium	[88]
*Epicoccum nigrum*	Mycoparasitism	[98]
*Phomopsis* sp. CAFT69	Production of metabolites, excelsional etc.	[89]
*Trichoderma harzianum* T39	Induce the expression of defenses genes PR-2, PR-4, OSM-1, etc.	[80,101]
*Trichoderma harzianum* TriH_JSB36	Induce the activities of the defense enzymes, peroxidase, etc.	[81]
*Trichoderma asperellum* ICC012/*Trichoderma gamsii* ICC080	Induce the production of jasmonic acid	[83]
*Paenibacillus* sp. B2	Production of paenimyxin, an antagonistic peptide	[73]
*Pseudomonas fluorescens* PTA-CT2	Induce the expression of defenses genes in SA and HR response	[71]
*Lysobacter capsici AZ78*	Production of cyclo (L-Pro-l-Tyr)	[77]
*Streptomyces atratus* PY-1	Production of metabolites (5-acetoxycycloheximide and cycloheximide)	[63]
*Streptomyces* sp. ANK313	Production of metabolites (Khatmiamycin)	[64]
*Bacillus subtilis* GLB191	Production of cyclic lipopeptides fengycin and surfactin; secretion of autolysins	[54,102]
*Bacillus megaterium* BMJBN02	Induce the content of salicylic acid and expression of defenses genes (PR genes)	[58]
*Bacillus velezensis* KOF112	Induce the expression of defenses genes (class IV chitinase and β-1,3-glucanase)	[59]

### 4.1. Mycoparasitism

Some biocontrol agents have the ability to mycoparasitize *P. viticola* and thereby control grape downy mildew. Through microscope observations, it was found that *F. proliferatum* G6 could coil around and inside the sporangiophores of *P. viticola* [84]. The mycelia of another *Fusarium* strain, *F. proliferatum* F3, was found to be able to coil around or cover the sporangiophores of *P. viticola*, including the entire downy mildew layer [85]. Five species of Fusarium species coil and induce the lysis of P. viticola sporophores [87]. Compared with plants not infected with *P. viticala*, in the *P. viticala*-infected group, the hyphae of *E. nigrum* rapidly surrounded the sporangiophores of *P. viticala* [98].

### 4.2. Competition

Competition with pathogens for nutrients or ecological niches is also an important control mechanism of biocontrol agents. Jaber [92] found that *Beauveria bassiana* ATCC 74040 exhibited the strongest colonization ability compared with all other tested strains, which might compete for plant ecological niches or resources with *P. viticola* and thereby confer protection against grape downy mildew. Musetti et al. [91] found that space and/or nutrient competition might be involved in *A. alternata* and *P. viticola* interaction.

### 4.3. Secretion of Enzymes and Peptides

Degradative enzymes or peptides secreted by biocontrol agents exert direct effects on *P. viticola*. Wang et al. [102] disrupted the gene LytD in *B. subtilis* GLB191, which encodes the autolysin N-acetylglucosaminidase—an endogenous cell wall-degrading enzyme in *B. subtilis*. The gene-disruption mutants reduced the suppression of *Plasmopara viticola*, in addition to the stimulation of plant defense. An antagonistic peptide, paenimyxin, was isolated and purified from *Paenibacillus* sp. strain B2, which was found to be able to inhibit the motility of *P. viticola* zoospores [73]. Other cyclic lipopeptides such as fengycin and surfactin produced by *B. subtilis* GLB191 have contributed to the control of grapevine downy mildew [53]. *Lysobacter capsici* AZ78 is able to produce cyclo(L-Pro-l-Tyr), which is toxic to the sporangia of *P. viticola*. Regarding fungal biocontrol agents, cyclo (L-phenylalanine-trans-4-hydroxy-l-proline), cyclo (L-leucine-trans-4-hydroxy-l-proline), and cyclo (L-alanine-trans-4-hydroxy-l-proline), isolated from *A. alternata,* were able to reduce the disease severity of grape downy mildew caused by *P. viticola* [88,90].

### 4.4. Production of Metabolites

Secondary metabolites produced by biocontrol agents have an effect on *P. viticola*. In a study, excelsional, alternariol, and 9-hydroxyphomopsidin produced by *Phomopsis* sp. CAFT69 showed strong motility inhibitory and lytic activities against the zoospores of *P. viticola* [89]. Metabolites produced from *Streptomyces* were found to follow a similar mechanism to *Phomopsis* sp. CAFT69. Khatmiamycin produced by Streptomyces sp. ANK313 was able to exert a complete motility inhibition effect on *P. viticola* and was also able to lyse the zoospores of *P. viticola* [64]. In other *Streptomyces* strains, 5-acetoxycycloheximide and cycloheximide produced by *S. atratus* PY-1 were significantly effective against *P. viticola* [63]. For fungal biocontrol agents, secondary metabolites such as fusaric acid produced by several *Fusarium* species, including *F. delphinoides* M1, *F. brachygibbosum* M2, *F. pseudonygamai* M10 and M12_1, and *Fusarium* sp. M12_2, were able to inhibit the growth of *P. viticola* [86,87]. Pen extracted from the dry mycelium of *Penicillium chrysogenum* was found to be able to reduce the disease severity of grape downy mildew [88].

### 4.5. Induction of Plant Systemic Resistance

The induction of plant systemic resistance is an important mechanism for controlling grape downy mildew. In a study, several plant defense response-related genes in grape plants were induced and expressed after being treated with biocontrol agents. The content of salicylic acid and the expression levels of defense-related genes (PR1, PR2, PR5, and PR10.2) were induced in grape plants after being treated with *B. megaterium* BMJBN02 against grape downy mildew [58]. Plant defense response-related genes encoding class IV chitinase and β-1,3-glucanase were upregulated in grape leaves treated with *B. velezensis* KOF112 [59]. Similar findings have been reported in *Pseudomonas fluorescens*. The application of *Pseudomonas fluorescens* PTA-CT2 can induce the systemic resistance of grape plants to *P. viticola* infection. The expression of defense genes, such as salicylic acid, and hypersensitive response-related genes were induced following the usage of *P. fluorescens* PTA-CT2 [71]. In fungal biocontrol agents, the expression levels of genes encoding pathogenesis-related protein 2 (PR-2), pathogenesis-related protein 4 (PR-4), and osmotin 1 (OSM-1) were increased in grape plants after the application of *T. harzianum* T39 [80]. The application of *T. harzianum* TriH_JSB36 increases the activities of defense enzymes in grape plants, including phenylalanine ammonia-lyase, peroxidase, and 1,3-glucanase [81]. Jasmonic acid production was induced in plants inoculated with *P. viticola* when the plants were treated with Trichoderma asperellum ICC012/Trichoderma gamsii ICC080 [83]. Yacoub et al. found that *Pythium oligandrum* Po37 can induce the expression of grapevine defense genes (PR1, GLU, PR5, LOX2, PAL, and STS) against *P. viticola* attack [94].

## 5. Conclusions and Future Prospects

Grape downy mildew is a type of airborne disease caused by *P. viticola* that can cause significant grape yield decline and severe economic losses. As a green, sustainable, and environmentally friendly control method, biocontrol has attracted considerable attention. Despite there being a plethora of studies on biocontrol agents, microbial resources have seldom been reported to be involved in controlling grape downy mildew. At present, the reported biocontrol resources capable of controlling grape downy mildew mainly involve bacteria such as *Bacillus*, *Streptomyces*, *Pseudomonas*, *Paenibacillus*, and *Lysobacter,* and fungi such as *Fusarium*, *Beauveria*, *Saccharomyces*, *Trichoderma*, and *Pythium*. Therefore, this review focuses on a detailed introduction to the biocontrol agents that could be used to effectively control grape downy mildew, together with their potential control mechanisms. This review provides a basis for the more efficient use of biocontrol agents in controlling grape downy mildew.

Future research can be carried out by exploring biocontrol microbial resources, including screening novel biocontrol agents, improving the control efficiency of existing biocontrol agents, constructing biocontrol genetic engineering strains, developing biocontrol agent products, and elucidating the molecular mechanisms involved in the control mechanism of biocontrol agents and the pathogenic mechanism of *P. viticola*.

(1) Screening for novel biocontrol microbial resources: There are currently still too few microbial resources able to control grape downy mildew. Novel biocontrol resources can be isolated from the soil, rhizosphere, or grape tissues using traditional isolation and purification methods, followed by being screened to determine their biocontrol efficacy. Alternatively, known microbial resources with broad-spectrum antimicrobial properties can be used to evaluate their control capabilities against grape downy mildew.
(2) Improving the control efficiency of existing biocontrol agents: Improvement mainly involves optimizing cultivation or application conditions, in addition to improving the ability of biocontrol agents to produce enzymes and secondary metabolites. Moreover, a synergistic combination of different biocontrol agents, or the integration of biocontrol agents with pesticides, will further improve control efficiency.
(3) Constructing biocontrol genetic engineering strains: The transformation of effective biocontrol genes into biocontrol agents to construct genetic engineering strains, thereby improving biocontrol efficiency against grape downy mildew.
(4) Elucidating the control mechanism of biocontrol agents: Clarifying the control mechanism of biocontrol agents is crucial for further improving the control efficiency of biocontrol agents. Transcriptomics can be used to screen biocontrol-related genes in biocontrol agents that are significantly differentially expressed during the process of controlling grape downy mildew. Thereafter, gene knockout, silencing, or overexpression could be used to verify the function of differentially expressed genes in controlling grape downy mildew.
(5) Elucidating the pathogenic mechanism of *P. viticola*: Clarifying the pathogenic mechanism of *P. viticola* could provide potential targets for biocontrol agents. Transcriptomics can be used to screen the differentially expressed genes during the infection process of *P. viticola* on grape tissues; thereafter, the function of these differentially expressed genes in *P. viticola* infection can be studied.
(6) Developing biocontrol products: Although some biocontrol agents have been reported, very few biocontrol agents have been developed into commercial products and widely used in field control. Therefore, the development of more commercial biocontrol products and the improvement of their shelf life will be of great value for the sustainable application of biocontrol agents.

## Data Availability

Not applicable.
